# Hypoxia as challenge and opportunity: From cells to crops, to synthetic biology

**DOI:** 10.1093/plphys/kiae640

**Published:** 2024-12-06

**Authors:** Julia Bailey-Serres, Peter Geigenberger, Pierdomenico Perata, Rashmi Sasidharan, Markus Schwarzländer

**Affiliations:** Center for Plant Cell Biology and Department of Botany and Plant Sciences, University of California, Riverside, CA 92521, USA; Plant Stress Resilience, Department of Biology, Utrecht University, 3584 CH Utrecht, The Netherlands; Faculty of Biology, Ludwig-Maximilians-University Munich, 82152 Planegg-Martinsried, Germany; PlantLab, Institute of Plant Sciences, Sant’Anna School of Advanced Studies, 56010 Pisa, Italy; Plant Stress Resilience, Department of Biology, Utrecht University, 3584 CH Utrecht, The Netherlands; Institute of Plant Biology and Biotechnology (IBBP), University of Münster, 48143 Münster, Germany

## Introduction

Oxygen is essential for plant survival. It supports a wide array of metabolic processes, including respiration. Hence, limitations in oxygen availability can pose a serious threat to plant life. Hypoxia can occur due to various environmental factors, such as flooding or ice encasement, pathogen infection, and is also intrinsic to high altitude. Hypoxia triggers a complex cascade of molecular and physiological responses within plants. Understanding these responses is crucial for developing strategies to improve plant tolerance to low-oxygen conditions. While the discovery of the plant cysteine oxidase (PCO)-N-degron pathway of oxygen sensing, a ubiquitin-mediated proteolysis pathway that plays a crucial role in regulating plant responses to hypoxia, remains at the center of the current research, two major questions have been drivers of recent discoveries: (i) how oxygen sensing is integrated with other stimuli and signals to modulate the acclimation responses of the plant and (ii) how the broader environmental and developmental context shapes the hypoxia response and adaptive characteristics of plant cells. A major proportion of our mechanistic understanding is still derived from Arabidopsis and rice as models; yet the diversification of the hypoxia sensing pathway through response programs in different crops is opening new avenues for fundamental research and for translation toward crop improvement. Experimental systems and the toolkit of research methods to study hypoxia responses have seen major advances in recent years, and even synthetic approaches to modify the relevant pathways have been pioneered. This Focus Issue aims to synthesize the latest developments and the emerging view of how plants sense and respond to hypoxia. Nine Updates and one Topical Review discuss key advances that have been made by a very active global research community over the last years. In addition, several research articles in this Focus Issue as well as previous issues of *Plant Physiology* add specific new research insights. The articles examine the genetic and molecular mechanisms that underpin flexible to enduring processes, with a particular focus on the role of specific genetic players and signaling pathways. One key area of focus remains the PCO-N-degron pathway and its role in oxygen-dependent degradation of specific proteins, including the Group VII Ethylene Response Factors (ERF-VIIs)—transcription factors that activate the expression of hypoxia-responsive genes. Yet, far from being a linear, single-purpose pathway, the articles of this issue explore the intricate interplay between hypoxia signaling and other environmental and developmental cues, such as hormonal signaling, nutrient and energy availability, and seed development and germination. The importance of metabolic acclimation, such as recent insights into the regulation of glycolysis and fermentation are covered, the role of reactive oxygen species (ROS), and the contribution of different cell organelles, such as mitochondria and the endoplasmic reticulum, as sites where oxygen is consumed at high rates to maintain cellular function. This Focus Issue reflects the biological and methodological breadth that is currently applied by researchers to understand hypoxia in plants. We consider the exploration of hypoxia in plants as a focal point, to drive our fundamental understanding of how plants work, but similarly to drive real-world solutions for sustainability and food security.

## Strategies of plant hypoxia signaling and acclimation

A breakthrough in plant hypoxia research was the discovery of the oxygen-sensing pathway mediated by the PCO-N-degron pathway. In their Update, Fuentes-Terrón et al. (2024) explore how the Cys/Arg branch of the N-degron pathway controls oxygen sensing in plants. The authors examine the functional and structural characteristics of Methionine Aminopeptidases (MetAPs) in catalyzing Met cleavage and PCOs in catalyzing the subsequent Cys oxidation. Particular attention is given to their interactions with substrates, with a special focus on the ERF-VIIs, known to drive transcriptional responses to hypoxia. Additionally, Vernalization 2 and Little Zipper 2 proteins, which are linked to responses to developmental hypoxia and represent 2 additional targets of the PCO-N-degron pathway, are discussed. The authors argue that the evolution of MetAPs and PCOs across the plant kingdom underscores their critical role in regulating the early stages of this branch of the N-degron pathway.

While the PCO-N-degron pathway is central to hypoxia sensing, it is evident that it does not act in isolation. Rather, it is integrated into a signaling network with varied stimuli as inputs, and feedback and crosstalk mechanisms that we are just beginning to understand. Renziehausen et al. (2024), in their Update, examine the roles of energy, redox, and hormonal signaling pathways in modulating plant responses to hypoxia. Hypoxia-associated energy deprivation and carbon starvation are discussed with a view on the established energy signaling pathways via 2 highly conserved kinases—sucrose nonfermenting-related kinase 1 and target of rapamycin kinase. The causes of the drastic hypoxia-associated redox changes are discussed and linked to plasma-membrane-localized NADPH respiratory burst oxidase (RBOH) activity and the mitochondrial respiratory chain. The authors explore how ROS and reactive nitrogen species signals may modulate retrograde signaling pathways and MAP kinase signaling to crosstalk with energy signaling and hormonal regulation. The roles of phytohormones, particularly ethylene, are discussed in fine-tuning the hypoxia response to control both development and stress acclimation. An updated list of hypoxia-responsive genes of Arabidopsis is featured as part of the review. Since the original version of this list produced by Mustroph et al. (2009) has proven immensely useful to the plant hypoxia research community, it is reasonable to expect that this update will be a valuable resource to facilitate future research.

An increase in cytosolic free calcium was observed nearly 3 decades ago (Subbaiah et al. 1994; Sedbrook et al. 1996) and has since been believed to act as a key signal in the hypoxia regulatory network. Yet, the identity of the channels and transporters involved in shaping hypoxia-associated calcium dynamics has been unclear. Also, to what extent calcium signaling interacts with the hypoxia signaling network, most importantly, the PCO-N-degron pathway, at a mechanistic level remains to be elucidated. Similarly, the role that calcium plays as a regulator of metabolic enzymes in the reprogramming of metabolism during hypoxia has remained patchy. An Update by Bakshi and Gilroy (2024) explores the role of calcium signaling in the hypoxic response and provides a neat overview. It discusses the calcium transporters and channels that may plausibly be involved in choreographing calcium dynamics during hypoxia, including the cation proton exchanger and autoinhibited calcium-ATPase families. It then examines downstream responders such as calmodulin-like proteins and calcium-dependent protein kinases (CDPK), which may orchestrate metabolic and transcriptional adjustments to aid the hypoxia response of plant cells. The mechanistic basis of the crosstalk of calcium signaling with other signals, such as pH changes, ROS, and NO, is identified as a critical emerging question. Specifically, RBOH-mediated signaling is highlighted as integration point between cytosolic calcium signaling and ROS signatures. Finally, the interplay of calcium dynamics between cell compartments, such as between the cytosol and the mitochondria, is highlighted as a fertile emerging research direction.

A specific example of the interplay of calcium and ROS is the development of aerenchyma and adventitious roots. Aerenchyma and adventitious roots are 2 well-studied adaptive traits that contribute to improved internal oxygen diffusion in flooded plants. Both involve programmed cell death triggered by flooding-induced ethylene accumulation and downstream activation of ROS production via NADPH oxidases. Aerenchyma formation involves the targeted lysis and death of root cortical cells, whereas adventitious root emergence necessitates the death of epidermal cells overlaying adventitious root primordia. Yamauchi et al. (2017) previously demonstrated that in rice, inducible aerenchyma formation requires ROS production mediated by RBOHH. In this issue, they demonstrate the involvement of calcium signals and the rice CDPK5 and CDPK13 in RBOHH activation (Li et al. 2024), which is further highlighted by a News & Views article (Kunkowska 2024). The expression of these *CDPKs* strongly correlates with ROS production and cell death in specific cortical cell layers. RBOHH phosphorylation sites were identified as essential for activation by CDPK. Accordingly, *cdpk5cdpk13* double mutants are severely or moderately impeded in their ability to trigger aerenchyma or adventitious root production, respectively.

In their Update article, Gibbs et al. (2024) provide a comprehensive overview of the epigenetic, transcriptional, translational, and posttranslational mechanisms governing plant responses to low-oxygen stress. The role of posttranslational modifications, such as phosphorylation, secondary messengers, transcriptional cascades, and retrograde signaling from mitochondria and the endoplasmic reticulum in modulating transcription factor activity, and the expression of hypoxia-responsive genes are examined. The authors also explore epigenetic mechanisms, and the role of mediators involved in the low-oxygen response as well as the cotranscriptional and posttranscriptional processes that fine-tune mRNA translation to ensure effective gene expression during hypoxic conditions.

A major output of hypoxia signaling and priming is the reprogramming of metabolism in a way that it can maintain vital cellular and ultimately organismal functions. The Update by van Veen et al. (2024) provides an overview of the metabolic strategies exploited by plants to deal with flooding and low oxygen levels. The review offers a mechanistic framework highlighting key genetic and physiological components underlying hypoxic metabolism. Focusing on recent advances in hypoxia metabolism research, the authors highlight emerging concepts of hypoxia-like metabolic modes in specific tissues and organs even under aerated conditions. The role of a differentiated, high-resolution understanding of hypoxia metabolism improving plant resilience to a changing climate is discussed.

## Hypoxia as a driver and a result of plant evolution and development

Since the discovery of the PCO-N-degron pathway of oxygen sensing, an impressive body of work has clarified the molecular and biochemical pathway components. It has also underscored the importance of the pathway substrate—the ERF-VII transcription factors as vital for hypoxia acclimation in different environmental contexts. This is the focus of the Update authored by Holdsworth et al. (2024). They provide an overview of the impact of geography, altitude, and agriculture on the evolution of oxygen sensing in plants, focusing on adaptations in the extensively studied model systems *Arabidopsis thaliana* and rice. The power of tools like environmental genome-wide association studies (eGWAS) for characterization of genetic variation driving adaptation to distinct environments is highlighted, with eGWAS implicating loci such as the known ERF-VII player (RAP2.12) and the previously unassociated MED25 BINDING RING-H2 PROTEIN 1 (MBR1). MBR1 encodes a ubiquitin-protein ligase that regulates MEDIATOR25 (MED25) (Schippers et al. 2024), which interacts with two ERF-VII, namely RAP2.2 and RAP2.12 to regulate hypoxia responses (Castellana et al. 2024). The authors make a case for how such studies provide critical insights into molecular mechanisms underlying adaptation to extreme precipitation and high altitude and offer strategies for breeding crops resilient to climate-induced stressors like flooding.

While geography, altitude, and agriculture have left a deep mark in the evolution of low oxygen responses, so have the different developmental stages of plants. Seeds not only ensure the propagation of many plants and provide a major proportion of the calories needed by humankind; they have also turned out to be hotspots of oxygen-dependent regulation. An Update by Rolletschek et al. (2024) examines how endogenous hypoxic conditions emerge in seeds and how oxygen availability impacts seed development and germination. In that context, the intriguing question is discussed if more or less oxygen would be beneficial for quiescent seeds. The authors synthesize recent progress to identify morphological features that influence oxygen diffusion into and within seeds, strategies of active management of the hypoxic endogenous environment, and key genes and metabolic adaptations that underpin seed cell physiology under hypoxia. An intriguing role of endogenous hypoxia in pacing seed development rates is discussed, along with more established roles such as controlling dormancy, and germination success, particularly the strategy used by rice to elongate a hollow coleoptile to enable establishment in a flooded environment. Finally, the potential role of seed microbiota in modulating hypoxia tolerance is explored as an emerging research avenue with likely relevance for improving hypoxia tolerance and yields of crops.

Hypoxic zones develop not only in seeds but also in bulky organs such as fruits. Yet, the functional implications for hypoxia pre- and postharvest remain insufficiently understood. In a recent issue of *Plant Physiology*, Xiao et al. (2024) explored whether and how hypoxia occurs in ripening tomatoes, focusing on tissue-specific respiration, and gas diffusion. By combining magnetic resonance imaging, microcomputed tomography, and respiration kinetics, a 3D reaction–diffusion model is presented and validated using oxygen microsensors. The study reveals that oxygen gradients develop within tomato fruits, with locular tissues (gel and seeds) being the most hypoxic due to high respiration rates and diffusion resistance. The pedicel scar serves as the main oxygen entry point, while the skin limits gas exchange. As hypoxia shifts metabolism to fermentative respiration, ethylene production and thus ripening processes are impacted. These findings suggest that hypoxia in tomatoes plays a role in development, influencing ripening, and seed maturation, thus setting the stage for further exploration and applications in production and postharvest management of tomatoes and other produce.

Rice has become the textbook example for 2 distinct survival strategies: underwater escape and submergence quiescence. In their Topical Review, Ashikari et al. (2024) provide both historical and recent insights into the molecular factors determining growth and metabolic mechanisms that underpin these strategies that arose in ancient Oryza. The review showcases recent advancements in regulatory networks that provide resilience to flooding in rice. These discoveries are discussed in the context of their continued value for rice production and their translation to other crops.

Rice has also been the subject of molecular analysis of extracellular lipid barriers to water and gas diffusion typical of semi-aquatic plants. One barrier is the hydrophobic waxy cuticle that contributes to the formation of a gas film that envelopes submerged leaves. Disruption of cuticular wax production and deposition, as observed in the *leaf gas film 1/hydrosteroid dehydrogenase 1* (*lgf1/hsd1*) mutant of rice (Zhang et al. 2016) also reduces levels of underwater photosynthesis in submerged leaves (Kurokawa et al. 2018). Another important barrier is suberin that can form 1 or more cortical cell layers of roots. Suberin regulates nutrient and water transport, and oxygen retention. In roots of rice cultivated in severely hypoxic floodwaters, suberin monomers produced by differentiated exodermal cells are transported to the apoplastic space to form complex lipid polyester lamellae directly beneath the epidermis that limits radial oxygen loss (ROL). This barrier enables oxygen to diffuse from aerial tissues to meristematic cells at root tips. In this collection, Jiménez et al. (2024) report that *lgf1* is compromised in the formation of the ROL, not due to a lack of suberin, but due to the loss of a layer of glycerol esters that may reinforce the suberized apoplast. Rescue of the *lgf1* restores the presence of glycerol esters and the tight ROL barrier. Previously, Shiono et al. (2022) reported that abscisic acid induces exodermal suberin production in waterlogged adventitious roots. In a new article, Shiono et al. (2024) hypothesize that low levels of nitrate might trigger ROL barrier formation. Their results reveal that nitrate deficiency is sufficient to induce the formation of suberin lamellae in the exodermis, coinciding with ROL barrier formation and demonstrating that extremely low nitrate levels serve as a critical environmental signal for initiating ROL barrier formation. Nitrate reduction triggered by prolonged waterlogging could therefore represent the key signal inducing ROL barrier formation in rice.

Besides ROL barrier development, shoot-borne adventitious root formation is part of the responses to waterlogging in many species. Adventitious root production can be inhibited by jasmonic acid, but a detailed mechanistic understanding of how JA regulates adventitious root formation under waterlogging stress remains elusive. Pan et al. (2024) discovered that JASMONATE ZIM-domain protein *Cs*JAZ8 plays a key role in adventitious root production in waterlogged cucumber (*Cucumis sativus* L.) plants. Overexpression of *CsJAZ8* inhibited waterlogging-induced adventitious root formation, providing further evidence of the involvement of jasmonate in this process. Furthermore, *Cs*JAZ8 and *Cs*MYB6 were shown to interact, leading to inhibition of *Cs*sMYB6. Silencing of *CsMYB6* indeed promoted adventitious root formation, whereas *Cs*MYB6 was shown to bind the promoters of *CsACO2* and *CsGA20ox2*, enhancing their transcription. The authors highlight that the transcriptional cascade involving *Cs*JAZ8 and *Cs*MYB6 interaction negatively impacts cucumber waterlogging tolerance by inhibiting ethylene and gibberellin accumulation, thereby suppressing adventitious root formation.

Roots of plants in waterlogged soils experience severe hypoxia. Paradoxically, waterlogging triggers many symptoms reminiscent of drought, such as leaf dehydration and stomatal closure. Haverroth et al. (2024) construct a detailed timeline of how shoot physiology and hydraulics are impacted during and after waterlogging in common bean (*Phaseolus vulgaris*) comparing it to drought. Waterlogging disrupts water transport in leaves and stems of *P. vulgaris*, causing dehydration, stomatal closure, and xylem embolism. These changes create a hydraulic disconnection that protects stems but exacerbates leaf damage and dehydration. Further, dehydration-independent factors, such as chemical signals and ROS, might drive early hydraulic impairments and damage during waterlogging. This work underscores the complexity of plant responses to water extremes.

## Relevance of hypoxia responses in cross-kingdom interactions

Hypoxia-stabilized ERF-VIIs are also relevant in the context of plant interactions with other organisms. For instance, these transcription factors act within the hypoxic niche of nitrogen-fixing nodules of legumes such as *Medicago truncatula* to sustain nodulation capacity and nitrogen fixation (Rovere et al. 2023). The research article by Deng et al. (2024) exposes unanticipated involvement of hypoxia in soybean cyst nematode resistance. Hypoxia response genes are upregulated at the site of *Heterodera glycines* infection due to stabilization of ERF-VIIs. Remarkably, ERF-VIIs directly activate transcription of genes encoding pathogenesis-related proteins at infection sites, thereby contributing to immunity to cyst nematodes. Hypoxia is also relevant in the context of pattern-triggered immunity, which was reported in a recent issue of *Plant Physiology.* Mooney et al. (2024) observed that hypoxia in Arabidopsis serves to repress the PTI response, i.e. gene expression, MAPK signaling, and callose deposition, as activated by the bacterial elicitor flagellin 22. Interestingly, this repression was found to be largely independent of ERF-VIIs, but draws up parallels with the situation in mammals where a repressive effect of hypoxia on innate immunity is well documented. Similar trade-offs between plant defense and hypoxic responses were observed for plant–fungal interactions. The study by Brunello et al. (2024) finds that the plant transcription factor ORA59 represses the ERF-VII-mediated induction of hypoxia-responsive genes in the response of *A. thaliana* to *Botrytis cinerea* infection. Strikingly, the activation of ethanol fermentation as an important metabolic response controlled by ERF-VIIs is demonstrated to be detrimental to Botrytis tolerance. In addition to the contribution of ORA59 to plant defense, the study pinpoints a role for ORA59 in managing the posthypoxic reoxygenation phase. These studies highlight how deeply hypoxia-associated signaling and responses are intertwined with plant biotic interactions. Considering this degree of interaction will be highly relevant to future efforts toward improving crop resilience and agricultural practices.

## Tools and synthetic biology to understand and modify plant hypoxia responses

A major bottleneck in plant hypoxia research has been the lack of tools to characterize spatiotemporal oxygen dynamics in anatomically diverse plant tissues and various environmental conditions. Considerable advancements in this area offer hypoxia researchers an impressive palette of tools for oxygen measurement, visualization, and manipulation. These have spurred research in new directions, enabling discoveries of additional facets of hypoxia, notably the existence and functional relevance of chronic hypoxic niches, such as in the shoot meristems. Panicucci et al. (2024) provide a comprehensive update of currently available and emerging tools for hypoxia studies in plants, including the modeling approaches developed by Xiao et al. (2024), in this collection. The advantages and limitations of these approaches are discussed together with pressing outstanding questions that can now be pursued thanks to the availability of these tools.

In a separate Update, Lavilla-Puerta and Giuntoli (2024) focus on the utility of synthetic biology techniques to develop biosensors, genetically encoded circuits, and orthogonal systems that can monitor and control hypoxia responses at the cellular level. These tools, adapted from animal, yeast, and bacterial research, or newly designed for plants, allow precise control of oxygen-related processes. The authors identify potential challenges limiting adoption of synthetic biology approaches, such as hurdles to application in nonmodel species and the need for improved prototyping methods. Advancement of synthetic biology technologies and approaches will be critical both for advancement of our fundamental understanding of hypoxia sensing and signaling in plants, and equally to boost our efforts to improve plant resilience to hypoxia-related stresses in the context of global climate change and sustainability challenges.
